# Probiotics for the Management of Oral Mucositis: An Interpretive Review of Current Evidence

**DOI:** 10.34172/apb.2023.029

**Published:** 2022-01-05

**Authors:** Maryam Fallah, Negin Amin, Mohammed H. Moghadasian, Sadegh Jafarnejad

**Affiliations:** ^1^Research Center for Biochemistry and Nutrition in Metabolic Diseases, Kashan University of Medical Sciences, Kashan, I.R. Iran.; ^2^Department of Food and Human Nutritional Sciences and the Canadian Centre for Agri-Food Research in Health and Medicine, University of Manitoba, Winnipeg, Canada.

**Keywords:** Cancer, Chemotherapy, Mucositis, Oral mucositis, Probiotic, Radiotherapy

## Abstract

Mucositis is one of the major side effects of anti-cancer therapies. Mucositis may lead to other abnormalities such as depression, infection, and pain, especially in young patients. Although there is no specific treatment for mucositis, several pharmacological and non-pharmacological options are available to prevent its complications. Probiotics have been recently considered as a preferable protocol to lessen the complications of chemotherapy, including mucositis. Probiotics could affect mucositis by anti-inflammatory and anti-bacterial mechanisms as well as augmenting the overall immune system function. These effects may be mediated through anti microbiota activities, regulating cytokine productions, phagocytosis, stimulating IgA releasement, protection of the epithelial shield, and regulation of immune responses. We have reviewed available literature pertaining to the effects of probiotics on oral mucositis in animal and human studies. While animal studies have reported protective effects of probiotics on oral mucositis, the evidence from human studies is not convincing.

## Introduction

 Anti-carcinoma therapies have various side effects, including mucositis; mucositis may be developed in up to 80% of cancer patients.^1,2^ In this regard, the diversity and population of oral microflora, as well as subjects’ diets play a crucial role.^3^ Mucositis could appear as painful oral ulcers with potential gastrointestinal complications, such as diarrhea and nausea.^3^ Almost all cancer patients who undergo bone marrow transplant or myeloablative therapy are at risk for oral mucositis.^4^ As an unwanted complication of anticancer therapy, mucositis may lead to exacerbation of symptoms, especially in young patients with depression, infection, and pain.^5^ Several factors at the level of submucosa and epithelium contribute to the formation of oral mucositis.^6^ Although there is no specific treatment for oral mucositis, a number of pharmacological and non-pharmacological strategies are available to suppress its development.^7^ Low energy laser is one of these strategies.^8^ Recent evidence demonstrates the promising effect of natural agents, including probiotics in healing oral mucositis lesions.^9,10^ Probiotics are known to enhance the functions of gastrointestinal tract and immune system. Among probiotics, *Lactobacillus* and *Bifidobacterium* are the most common bacteria,^11^ and *Saccharomyces boulardii* is the best-known yeast.^12^ Beneficial roles of probiotics have been reported in a number of disorders, including various types of diarrhea, *H. pylori* inflammation, inflammatory bowel disease, inflammatory bowel syndrome, gluten intolerance, gastrointestinal cancer, and mucositis.^13^ Furthermore, probiotics may have psychological protective effects, reducing the risk for the development of depression, perceived stress, and anxiety.^14^ Benefits of probiotics in a rat model of chemotherapy-induced mucositis, and intestinal injuries have been reported.^15^ *Lactobacillus reuteri* is known to be a beneficial probiotic for peri-implant mucositis and *Lactobacillus brevis *CD2 is an effective species of probiotics for inhibiting chemotherapy-induced oral mucositis.^3^ Probiotics may lead to healing of oral mucositis by improving the immune system function.^16^ Also, they may increase the host’s defence mechanisms to overcome *Streptococcus mutans* by increasing the synthesis of immunoglobulins.^17^ Even though there has been a lot of attention to probiotics as adjuvant therapy for oral mucositis, the current evidence is not convincing.^3^ In this review, we have critically evaluated current literature on the benefits of probiotics in the management of oral mucositis along with their potential mechanisms of action.

## Pathogenesis of oral mucositis

 As a multifactorial disorder, mucositis is categorized into the oral and gastrointestinal types, based on the tissue it has damaged.^18^ The actual pathogenesis of oral mucositis is still under discussion. Damages to several types of cells, and tissues of the oral mucosa have been reported in oral mucositis.^19^ The functions of oral microorganisms in the treatment or prevention of this disorder are still unknown.^20^ One of the possible reasons that radiotherapy results in oral mucositis is its destructive effects on DNA molecules.^21^ As shown in a previous study, the thickness of the oral mucosa epithelium was minimized significantly after chemotherapy.^22^ The authors of this study suggested that oral mucositis lesions could be a toxic side effect of mammalian target of rapamycin (mTOR) inhibitors, like everolimus. Limited studies have reported benefits of steroids against mTOR-induced oral ulcers.^23^ Animal studies also reported that induction of mucositis was associated with changes in inflammatory pathways and nitric oxide metabolism.^24^ Available literature suggests that the transcription factor NF-κB plays a crucial role in the formation of mucositis.^25^ This may result in increasing cyclooxygenase-2 activity, leading to accumulation of submucosal fibroblasts and increased prostaglandin production. In cancer patients, an alteration in oral or intestinal bacterial microflora typically happens through the usage of antibiotics, xerostomia, and neutropenia. Also, after transplantation of hematopoietic cells, some microbiota (mostly from streptococcispecies) have been detected in the oral cavity. Other biofactors like TNF, IL-1B, MMP-3 and other inflammatory markers as well as epidermal growth factor may also play a role in the development of oral mucositis.

## Management of oral mucositis

 Despite advances in medical therapy, our knowledge in the area of prevention and treatment of drug-induced mucositis is very limited.^26^ Washing the oral cavity with saline associated with the use of soda bicarbonate, benzydamine, and low-degree laser are commonly recommended for the prevention of radiation-induced oral mucositis.^27^ Benzydamine hydrochloride (BZD) has multiple biological functions that can interfere with the processes of oral mucositis formation.^2^ It has been reported that BZD consumption could cause an increment in epithelial cell proliferation and a decrement in the secretion of inflammatory cytokines, like IL-1B and TNF-a.^28^ Low-level laser therapy (LLLT) may prevent the development of mucositis or reduce its severity, especially in younger patients.^8^ Co-administration of photochemotherapy and LLLT may result in synergic beneficial effects on oral mucositis status.^29^ Photodynamic therapy could be recommended for the treatment of mucositis in children and younger patients.^30^ Another method of treatment is using 0.5% methylene blue for washing the oral cavity. It could soothe the pain of oral mucositis ulcers.^31^ Oral cryotherapy is the other treatment for preventing and reducing the severity of chemotherapy-induced oral mucositis.^32^ In this method, practitioners chill the oral cavity by using ice, ice cream, or cold water to reduce blood flow and thereby reduce the local effects of the chemotherapy agents on oral mucosa.^33^ On the other side, Smad7 could suppress NF-κB and TGFβ, causing a decrease in apoptosis and inflammation while increasing epithelial migration. This could suggest that Smad7 could be considered as a major treatment for oral mucositis.^6^ Human keratinocyte growth factors, such as Palifermin could be also considered for the treatment of oral mucositis.^34^ Soft and liquid diets are suggested for patients with oral mucositis to ease eating and facilitate adequate nutrient intakes.^35^ Lately, more attention has been paid to the use of natural products, including honey, aloe vera, royal jelly, and propolis for their roles in the prevention and/or treatment of cancer-induced oral mucositis.^36^ Black mulberry molasses is another example of natural products used to reduce the burden of oral mucositis.^37^ Hydration, patient education, proper dietary intakes, and pain reduction methods are also included in the treatment protocols for oral mucositis. In addition to the above-mentioned protocols, several studies have reported the benefits of probiotics in the management of oral mucositis as discussed below.

## Probiotic and oral mucositis

###  Animal studies

 Probiotic supplementation alleviated oral and intestinal infection in a rat model of chemotherapy-induced mucositis.^15^ *Streptococcus thermophilus *TH-4 was recently introduced as a probiotic that improves chemotherapy-induced mucositis via the folate production-like pathway.^38^ Another study suggested that probiotics (*Lactobacillus* and *Bifidobacteria*) may activate anti-viral macrophages through the secretion of nitric oxide and inflammatory agents like IL-6.^39^ The effects of various probiotics on animal models of oral mucositis are summarized in Table 1.

**Table 1:** Characteristics of *In vivo* and *In vitro* studies depicting the effects of probiotics on oral mucositis and the complications

**Reference**	**Model**	**Treatment agent**	**Treatment course**	**Major outcome**
Gerhard et al^15^	Male Wistar rats (oral and intestinal mucositis induced by chemotherapy)	*Bacillus subtilis*, *Bifidobacterium bifidum*, *Enterococcus faecium* and *Lactobacillus acidophilus*	3-7 days	Improvement in immune response and reduction in oral and intestinal inflammation have been observed
Trindade et al^40^	5-Fluorouracil-induced induced male mice	*Lactobacillus paracasei*, *Lactobacillus rhamnosus*, *Lactobacillus acidophilus* and *Bifidobacterium lactis*	13 days	Mucositis damage has been reduced by synbiotic consumption
Wang et al^38^	Methotrexate induced mucositis female rats	*Streptococcus thermophilus* TH-4	8 days	Probiotic has prevented weight loss in samples but did not indicate any other therapeutic effect
Ivec et al^39^	In vitro	*Lactobacillus* or *Bifidobacteria*	24 h	Probiotics have shown antivirus effects
Mauger et al^41^	5-FU-induced intestinal mucositis female rats	*Lactobacillus fermentum* BR11, *Lactobacillus rhamnosus* GG, *Bifidobacterium lactis* Bb12	10 days	The probiotic beneficial effects were not significant
Huang et al^42^	5-FU-induced mucositis in BALB/c mice	*L. casei* variety *rhamnosus* and *L. acidophilus* and *B. bifidum*	5 days	Probiotic consumption led to improve cytokines level
Yeung et al^43^	5-FU-induced intestinal mucositis in mouses	*Lactobacillus casei variety rhamnosus*(Lcr35)or* Lactobacillus acidophilus *and* Bifidobacterium bifidum *(LaBi)	5 days	Probiotics have improvement effects on chemotherapy-induced mucositis
Song et al^44^	In vitro	*Lactobacillus rhamnosus*and* Lactobacillus casei*	30 days	These species have significant antifungal properties

###  Human studies

*Lactobacillus reuteri* and *L. brevis * CD2 have been shown to produce beneficial effects against chemotherapy-induced oral mucosa injuries.^3^ In a double-blind trial, the effects of these probiotics on peri-implant mucositis patients have been investigated. The results of this investigation indicated synergistic effects of oral hygiene and probiotics in alleviating symptoms of mucosal injuries.^45^ The beneficial effects of *L. rhamnosus, L. acidophilus,* and *B. bifidum *have been noticed in candidiasis patients. The probiotic product showed a reducing effect on the colonization of *Candida* in denture wearers.^46^ It seems that probiotics are more useful than usual antifungal therapies in ameliorating the prevalence and complications of candidiasis.^47^ The combination of mechanical therapies with probiotics (*L. reuteri*) seems to be more effective than only mechanical therapy for the implant and peri-implant mucositis treatment. Similarly, the use of *L. reuteri* alone has minimal effects on peri-implant microbiota.^48^ The effects of probiotics on peri-implant mucositis may be mediated through regulating cytokines and other biomarker levels.^49^ In a triple-blind clinical trial, the positive effects of probiotics (*L. reuteri )* on mucositis have been evaluated. Reductions in implant’s mucositis were associated with reduced *P. gingivalis* population in the oral cavity.^48^ Another study reported *L. rhamnosus* and *L. casei’s* anti-fungal function which could be helpful in candidiasis treatment.^44^ There wasn’t any noticeable change in oral microbiota after the consumption of probiotic drinks in healthy denture wearers.^50^ In a triple-blind study, *L. reuteri* was used for peri-implant mucositis treatment, but the outcomes were comparable among all study groups.^51^ Another examination on oropharyngeal mucositis did not report the benefits of *L. brevis CD2* in head and neck cancer patients. The benefits of probiotics on human mucositis are summarized in Table 2.

**Table 2:** A summary of human studies of the probiotic supplementation on oral mucositis

**Reference**	**Model**	**Treatment agent**	**Treatment course**	**Major outcome**
Flichy Fernández et al^49^	Peri-implant mucositis (n = 77)	*Lactobacillus reuteri*	30 days	Probiotic consumption led to improve clinical symptoms and cytokines level
Hallström et al^45^	Peri-implant mucositis (n = 49)	A mix of two strains of *Lactobacillus reuteri* (DSM 17938 and ATCC PTA 5289)	26 weeks	No significant advantage of probiotic consumption has been reported
Ishikawa et al^46^	Candida infection (n = 59)	*Lactobacillus rhamnosus* HS111, *Lactobacillus acidophilus* HS101, and *Bifidobacterium bifidum*	5 weeks	The combination of these 3 strains of probiotic has been useful for lowering the colonization of Candida in the oral cavity
Li et al^47^	Candida-associated stomatitis (n = 65)	*Biﬁdobacterium longum*, *Lactobacillus bulgaricus* and *Streptococcus thermophilus*	4 weeks	Noticeable improvement in some signs have been reported
Galofré et al^48^	Mucositis and pre-implantitis (n = 44)	2 strains of L. reuteri (ATCC PTA 5289, DSM 17938)	90 days	Probiotic consumption has demonstrated limited positive effects
Jiang et al^52^	CCRT induced oral mucositis (n = 99)	*Bifidobacterium longum*, *Lactobacillus lactis*, and *Enterococcus faecium*	7 weeks	Improvement in oral mucositis and immune response have been observed
Sutula et al^50^	Healthy complete denture wearers (n = 7)	*Lactobacillus casei* strain Shirota (LcS)	7 weeks	No significant change has been reported
Peña et al^51^	Implant induced mucositis (n = 50)	*L. reuteri (DSM 17938 and ATCC PTA 5289)*	3 months	No significant advantage of probiotic consumption has been reported
De Sanctis et al^53^	Radiotherapy induced oral mucositis in patients with head and neck tumor (n = 75)	*Lactobacillus brevis* CD2	4 weeks	No significant improvement has been observed

###  Possible mechanisms of the beneficial effects of probiotics on oral mucositis

**Figure 1:**
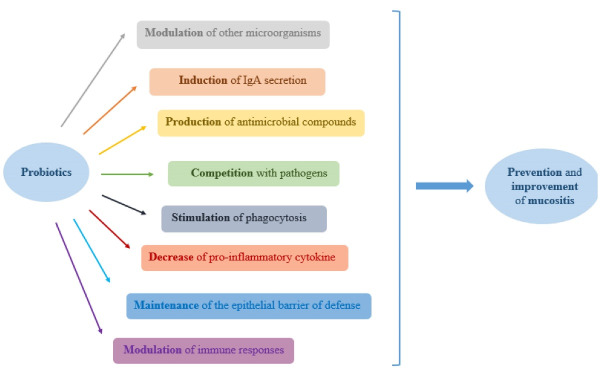
Suggested mechanisms for probiotics effects on oral mucositis.

 Probiotics might protect the mucosa from candida and other infectious agents through displacing different pathogens,^54,55^ regulating cytokine production and phagocytosis,^56,57^ stimulating IgA releasement, protection of the epithelial shield, and enhancing immune responses.^58,59^ It has been suggested that probiotics could stimulate the expression of anti-inflammatory agents like IL-1RII which binds to proinflammatory cytokines and neutralize them.^60,61^ The results of another study have shown that probiotics couldn’t make a significant impact on ameliorating oral scars when using lozenges and topical oils.^62^ It has been reported that *L. reuteri* DSM17938 and PTA 5289 could remove mutans of streptococci from the mouth cavity. *L. reuteri* might make a change on host genes and leads to variations in epitopes receptors.^63^ In vitro studies suggest two possible pathways for antiviral effects of probiotics. Probiotics could impede the virus and prevent absorption and cell internalization of the virus. Another possible way is that probiotics can communicate with cells to create an antiviral mechanism.^64^ Mechanisms by which probiotics may generate beneficial effects in the management of oral mucositis are illustrated in Figure 1.

## Conclusion

 In this review, we have reviewed and summarized information on the benefits of probiotics in the treatment and/or prevention of mucositis. The benefits of probiotics on alleviating complications of mucositis have been reported mainly through animal studies; such effects have not been produced by human studies.

 This could be because of limited number of human studies on the effects of probiotics on oral mucositis. Overall, it may be suggested that probiotics may generate beneficial effects on oral mucositis under certain conditions. However, more human studies are needed to establish the efficacy of different strains of probiotics on oral mucositis and their complications. It should be taken into account that different species of microbiota have their attributes and have specific mechanisms of action. Future studies should consider this fact and should examine the efficacy of different species of probiotics according to their specific mechanism and properties.^65^

## Acknowledgments

 We thank the Research Center for Biochemistry and Nutrition in Metabolic Diseases at Kashan University of Medical Sciences for the provision of facilities needed to perform this review article. MHM’s Research Program is supported by Natural Sciences and Engineering Research Council of Canada (NSERC).

## Competing Interests

 The authors do hereby declare that there are no actual or perceived conflicts of interest regarding this manuscript.

## Ethical Approval

 Not applicable.
